# The structure of salt marsh soil mesofauna food webs – The prevalence of disturbance

**DOI:** 10.1371/journal.pone.0189645

**Published:** 2017-12-14

**Authors:** Kristin Haynert, Mirijam Kiggen, Bernhard Klarner, Mark Maraun, Stefan Scheu

**Affiliations:** 1 J.F. Blumenbach Institute of Zoology and Anthropology, University of Göttingen, Göttingen, Germany; 2 Centre of Biodiversity and Sustainable Land Use, University of Göttingen, Göttingen, Germany; University of Sydney, AUSTRALIA

## Abstract

Mesofauna taxa fill key trophic positions in soil food webs, even in terrestrial–marine boundary habitats characterized by frequent natural disturbances. Salt marshes represent such boundary habitats, characterized by frequent inundations increasing from the terrestrial upper to the marine pioneer zone. Despite the high abundance of soil mesofauna in salt marshes and their important function by facilitating energy and carbon flows, the structure, trophic ecology and habitat-related diet shifts of mesofauna species in natural salt marsh habitats is virtually unknown. Therefore, we investigated the effects of natural disturbance (inundation frequency) on community structure, food web complexity and resource use of soil mesofauna using stable isotope analysis (^15^N, ^13^C) in three salt marsh zones. In this intertidal habitat, the pioneer zone is exposed to inundations twice a day, but lower and upper salt marshes are less frequently inundated based on shore height. The mesofauna comprised 86 species / taxa dominated by Collembola, Oribatida and Mesostigmata. Shifts in environmental disturbances influenced the structure of food webs, diversity and density declined strongly from the land to the sea pointing to the importance of increasing levels of inundation frequency. Accordingly, the reduced diversity and density was associated by a simplification of the food web in the pioneer zone as compared to the less inundated lower and upper salt marsh with a higher number of trophic levels. Strong variations in δ^15^N signatures demonstrated that mesofauna species are feeding at multiple trophic levels. Primary decomposers were low and most mesofauna species functioned as secondary decomposers or predators including second order predators or scavengers. The results document that major decomposer taxa, such as Collembola and Oribatida, are more diverse than previously assumed and predominantly dwell on autochthonous resources of the respective salt marsh zone. The results further suggest that Mesostigmata mostly adopt an intraguild predation lifestyle. The high trophic position of a large number of predators suggests that intraguild predation is of significant importance in salt marsh food webs. Presumably, intraguild predation contributes to stabilizing the salt marsh food web against disturbances.

## Introduction

Salt marshes are widespread along the European coasts and cover 20% of the area of the North Atlantic Wadden Sea [1]. The exposure to frequent inundations and the clear elevational gradient with the upper salt marsh, lower salt marsh and pioneer zone make them to ideal model systems to study, how physical and biological disturbance factors interact to create pattern in natural communities [2]. Although salt marshes provide important ecosystem services such as biomass production, supply of food sources, nitrogen and carbon cycling, they are also heterogeneous habitats, and plants and animals must cope with increasing abiotic disturbance due to increasing frequency of inundations towards the pioneer zone [3–7]. Depending on shore height, the pioneer zone is exposed to inundations twice a day [8], which are less frequent in the lower salt marsh with 150–250 times a year and the upper salt marsh with 35–70 times a year [9, 10]. The consequences of the harsh and dynamic abiotic environmental conditions results in typical vegetation zonation [10–13], which corresponds to a shift from terrestrial C3 to marine C4 plants/algae along the land sea gradient [14, 15]. Similar to the plant based zonation, the organic material of the soil differs between zones, depending on decomposition rates, the amount of deposition, and its origin from either marine or terrestrial resources. C3 and C4 material differ in their photosynthetic pathway resulting in distinct stable carbon isotopic signals allowing identification of their contribution as basal resources of food webs [16–19].

Intertidal communities historically played an important role in the development of community ecology since they occur across pronounced abiotic and biotic disturbed conditions along the land-sea gradient [20], encompassing a large number of microhabitats with varying niches on small spatial scales [21]. Disturbance is known to strongly determine ecological patterns and processes, affecting diversity and dynamics, as well as the food web structure of communities [6, 20, 22, 23]. Consequently, ecological settings are complex and largely control the isotopic structure of salt marsh soil food webs [10, 14, 24–26]. However, despite the central role of terrestrial soil mesofauna in facilitating energy and carbon flows between trophic levels, their structure, trophic ecology and habitat-related diet shifts in salt marsh food webs is virtually unknown.

The salt marsh soil community is strongly size structured and previous studies investigated distribution patterns [27–29], food web structure and feeding preferences of macro-invertebrates [14, 30–32] as well as the influence of autochthonous and allochthonous resource material on community structure [5, 33–35]. Macro-invertebrates comprise different feeding types allowing to utilize diverse food resources, accordingly their diet changes along the environmental gradient [32, 36, 37]. However, previous studies mostly focused on higher trophic levels and neglected the complexity and functional role of small mesofauna species such as Collembola, Oribatida and Mesostigmata.

Collembola and Oribatida are diverse and abundant and play multiple roles in salt marsh food webs [38, 39]. They may significantly affect bacterial density and biomass [40], are important decomposers and microbivores, thereby functioning as primary and secondary decomposers as well as predators and scavengers. Small predators such as Mesostigmata are among the most effective predators in soils and sediments [41], but knowledge of their trophic ecology is based primarily on laboratory observations with only few species studied in detail [42]. Further, mesofauna taxa are major food sources for macro-invertebrates [43–45]. The few studies existing suggest that mesofauna abundance and diversity differ substantially between salt marsh zones with the diversity being low in the lower salt marsh and increasing with plant cover at higher salt marsh zones [46–48]. Increased abundance and diversity is likely to affect food web structure with the number of trophic levels increasing with habitat productivity and resource availability [49].

In order to understand the effect of disturbances on mesofauna ecology in salt marshes, we investigated the mesofauna community structure, trophic levels and resource use of abundant species along a small-scale salt marsh gradient of the German North Sea using stable isotopes. The analysis of natural variations in stable isotope ratios is a powerful tool to study the trophic structure of soil animal communities [19, 50–52], and the flux of carbon from terrestrial and marine realms into animal food webs [53, 54]. We expected (1) soil mesofauna diversity and density to decrease from the upper salt marsh to the pioneer zone correlated with more frequent flooding and associated abiotic variations, (2) the reduced density and diversity to be associated with a simplification of the mesofauna food web in the pioneer zone as compared to the less disturbed lower and upper salt marsh with a higher number of trophic levels, and (3) dominant mesofauna species such as Collembola, Oribatida and Mesostigmata feeding at multiple trophic levels functioned as secondary decomposers or predators including second order predators or scavengers.

## Materials and methods

### Study site

**Fig 1:**
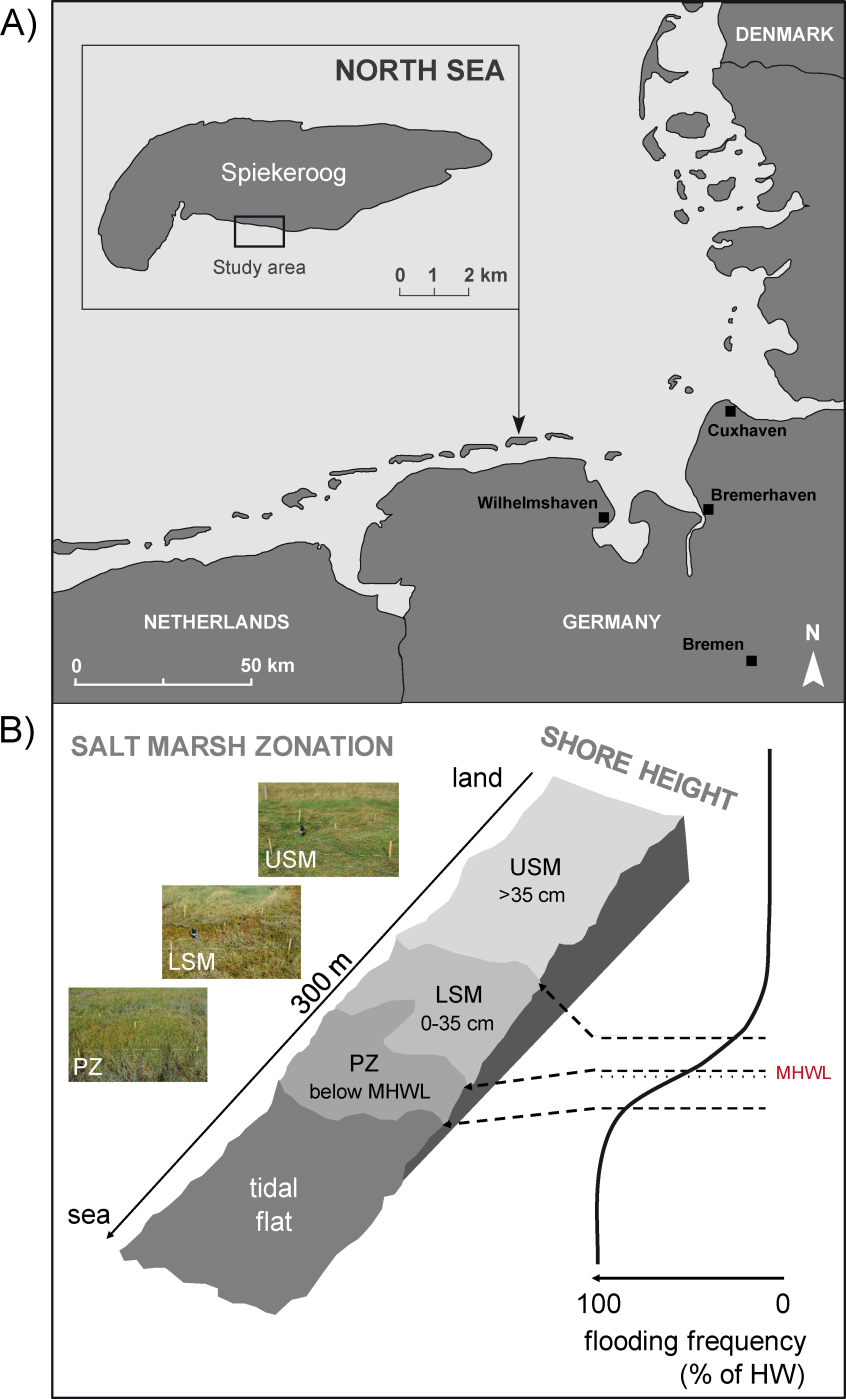
(A) Map of study area: Spiekeroog, East Frisian Islands, Germany, North Sea. (B) Salt marsh zonation: upper salt marsh (USM, >35 cm above MHWL), lower salt marsh (LSM, 0–35 cm above MHWL) and pioneer zone (PZ, below MHWL) from the land to the sea in relation to shore height and frequency of inundations (USM: 35–70 times a year, LSM: 150–250 times a year, PZ: inundations twice a day).

The study was performed in the salt marshes of the North Sea dune island Spiekeroog (53°45’2”- 53°47’1”N, 7°40’0”- 7°49’1”E), which belongs to the East Frisian Islands, forming part of the Wadden Sea National Park of Lower Saxony, Germany (Fig 1A). The Wadden Sea National Park of Lower Saxony gave the permission to conduct the study on this site, the current study did not involve endangered or protected species. Unlike many salt marshes along the German coastline, the salt marsh on Spiekeroog is not affected by extensive agricultural activities or urban development. Thereby, the island provides the opportunity to study the distribution and trophic structure of soil invertebrates under natural settings. Salt marshes are located in the southern part of the island, which is sheltered from northerly winds and incoming tides ranging 1 to 3 m. The boundary between terrestrial and marine habitats is characterized by halophytic plants close to the mean high water line (MHWL) and consists of three vegetation zones: upper salt marsh, lower salt marsh and pioneer zone (Fig 1B). The sampling plots were selected according to these vegetation zones depending on shore height (upper salt marsh: >35 cm above MHWL, lower salt marsh: 0–35 cm above MHWL, pioneer zone: below MHWL) and frequency of inundation (upper salt marsh: 35–70 times a year, lower salt marsh: 150–250 times a year, pioneer zone: inundations twice a day; Fig 1B). The dominant C3 plant of the upper salt marsh is *Elytrigia atherica*, whereas the lower salt marsh predominantly is colonized by two specialized C3 plant species, *Atriplex portulacoides* and *Puccinellia maritima*. Vegetation in the pioneer zone is dominated by the C3 plant *Salicornia stricta* and the C4 plant *Spartina anglica* (Fig A and Table D in S1 File). Furthermore, C4 macroalgae species such as *Enteromorpha* sp. and *Ulva lactuca* are widespread in the pioneer zone (Fig A and Table D in S1 File).

### Experimental design and sampling

Six sampling plots of 1.80 x 1.80 m (3.24 m^2^) were established in each of the three salt marsh zones (upper salt marsh, lower salt marsh and pioneer zone) in the frame of the BEFmate-project [55]. On 24^th^ September 2014, soil samples were collected from the upper 5 cm of each sampling plot using a soil corer (Ø 5 cm). Each sampling plot consisted of four subplots 90 x 90 cm (0.81 m^2^), two samples were taken in two of the four subplots resulting in four pooled samples per plot per vegetation zone (72 samples overall). To avoid pseudoreplication, the mean of these four samples was used for statistical analysis resulting in a total number of 18 samples. Soil cores were placed in plastic containers and transported to the laboratory for extracting the animals. Furthermore, at each of the three vegetation zones, vascular plants, macroalgae and organic matter were sampled by hand or with a spatula for analysis of potential food resources, resulting in 21 samples with three replicates each, in total 63 samples. The samples were stored in plastic bags at -10°C until drying and further processing.

### Sample processing

In the laboratory, living soil invertebrates were extracted using a high gradient heat extractor [56]. This extraction method is not suitable to extract nematodes, therefore nematodes were excluded from soil invertebrate analysis. Soil invertebrates were fixed in 70% ethanol and stored at -10°C until identification. Preservation in 70% ethanol little affects ^13^C signatures of animals [57], but ^13^C signatures vary with e.g., body size and cuticle thickness as well as life stage associated with changes in the proportion of fat reserves. To exclude these variations in δ^13^C signatures, we used specimens of similar body size and focused mainly on adult individuals.

For identification individuals were placed on glass-slides, analyzed to species or genus level and counted under a binocular eyepiece or microscope. Following identification and depending on the number of individuals, a minimum of two replicates for each dominant mesofauna species (Collembola, Oribatida and Mesofauna) was prepared for stable isotope measurements, in total 27 species. Soil invertebrates were dried at 40°C for 24 to 48 h and placed in a desiccator. Samples >10 µg dry mass were weighed on a fine scale (Cubis, Sartorius, Göttingen, Germany) and transferred into tin capsules. Large species (>400 µm) were fragmented and homogenized using mortar and pestle, whereas smaller mesofauna species (<400 µm) were bulked with a maximum of five specimens per sample (the final weight of the samples is given in supporting information Table B in S1 File). Food resources (vascular plants and macroalgae) were washed with tap water to remove sediment and smaller algae before drying at 40°C for 24 to 48 h. After drying, organic matter samples were passed through 250–2000 μm screen in order to remove larger organic debris, shells and stones. Resources were homogenized using a mortar and the fine powder was transferred into tin capsules. The capsules filled with soil invertebrates and food resources were closed and wrapped into pellets, placed into well plates and stored in a desiccator until stable isotope analysis.

### Stable isotope analysis

To estimate the trophic position of animals in the salt marsh food web, natural variations in stable isotope ratios (^15^N/^14^N and ^13^C/^12^C) were analyzed [50, 58]. δ^15^N values were used to delineate the trophic position of the species and to ascribe species to trophic levels we assumed a consistent enrichment of 3.4 δ units per trophic level [50, 59]. In contrast to ^15^N, trophic level fractionation of ^13^C is low and varies between -0.5‰ [60] and 1 ‰ [61]. Therefore, δ^13^C signatures of consumers resemble that of their diet and can be used to evaluate the sources of carbon of consumers if the isotopic signatures of the sources are different [50], i.e. organisms feeding on C3 plants can be distinguished from those feeding on C4 plants [62].

Stable isotope ratios were analyzed using a coupled system consisting of an elemental analyzer (for mesofauna: Euro EA 3000, EuroVector S.p.A, Milan, Italy; for plants: NA 1500, 2500, Carlo Erba, Milan, Italy) and a gas mass spectrometer (Finnigan Delta V Plus, Thermo Electron, Bremen, Germany). The mean standard deviation ranged between <0.1 and 0.2 ‰ [63]. Signatures of stable isotopes were expressed using the δ notation with δ*X* (‰) = [(*R*_sample_—*R*_standard_)/*R*_standard_] x 1000. *X* represents the target isotope of δ^13^C or δ^15^N (‰), *R* the heavy-to-light isotope ratios (^13^C/^12^C and ^15^N/^14^N) of samples and standard, respectively. Vienna Pee Dee River Belemnite (PDB) and atmospheric nitrogen served as primary standards for δ^13^C and δ^15^N, respectively. Acetanilide was used for internal calibration. Deviation of stable isotope ratios of animals and potential resources were expressed using the Δ notation representing the differences between the soil animal stable isotope ratio and the respective ratio of salt marsh resources.

Stable isotope signatures of the organic matter varied considerably at the three salt marsh zones (δ^13^C values of -25.9 ± 0.76‰, -29.9 ± 0.26‰ and -13.5 ± 0.06‰ for the upper salt marsh, lower salt marsh and pioneer zone, respectively; respective δ^15^N values of 4.6 ± 0.50‰, 10.2 ± 0.45‰ and 12.0 ± 0.04‰). To determine the trophic positions of soil invertebrates, stable isotope signatures were normalized to the mean of the organic matter signature of the respective salt marsh zone (calibration factors for δ^13^C of +2.8‰, +6.8‰ and -9.6‰ for the upper salt marsh, lower salt marsh and pioneer zone, respectively; respective calibration factors for δ^15^N of +4.3‰, -1.3‰, -3.0‰). Although normalizing stable isotope values to that of soil organic matter may inadequately reflect the baseline of the animal community, normalization to dead organic matter has been shown to considerably improve comparison of trophic positions of soil invertebrates across different habitats [42].

### Statistical analysis

Community structure of the three salt marsh zones were analyzed by canonical correspondence analysis (CCA) and non-metric multidimensional scaling (NMDS) using CANOCO 5 (Microcomputer Power, Ithaca, USA, 2012). The data set of the NMDS was subsequently used for discriminant function analysis (DFA) in STATISTICA 7.0. For the CCA and the NMDS, non-identified individuals were excluded and the data were log-transformed.

In case of significant DFA, analysis of variance (ANOVA) and Tukey's HSD (honestly significant difference) test were performed for soil mesofauna density and diversity along the salt marsh gradient. Further, to inspect differences in the number of trophic levels between salt marsh zones and the relationship between the δ^13^C values of basal resources from the land to the sea ANOVA was conducted using R statistical programming environment version 2.4.0 (R Development Core Team 2007). Prior to ANOVAs data were inspected for normality using Shapiro-Wilks test using STATISTICA 7.0 (StatSoft, Inc., Tulsa, OK, 2004); the test generally was not significant (p > 0.05).

To estimate the relative intake of potential food sources of consumers (Collembola and Oribatida), the Bayesian mixing model FRUITS 2.1.1 Beta (Food Reconstruction Using Isotopic Transferred Signals) was used [64]. Mean values for the stable isotopes of three food groups (C3 plants/algae, C4 plants/algae and organic material, which contained C3 and/or C4 plants/algae) were used to determine the relative contributions of basal resources to Collembola and Oribatida nutrition in the salt marsh.

## Results

### Community structure

**Fig 2:**
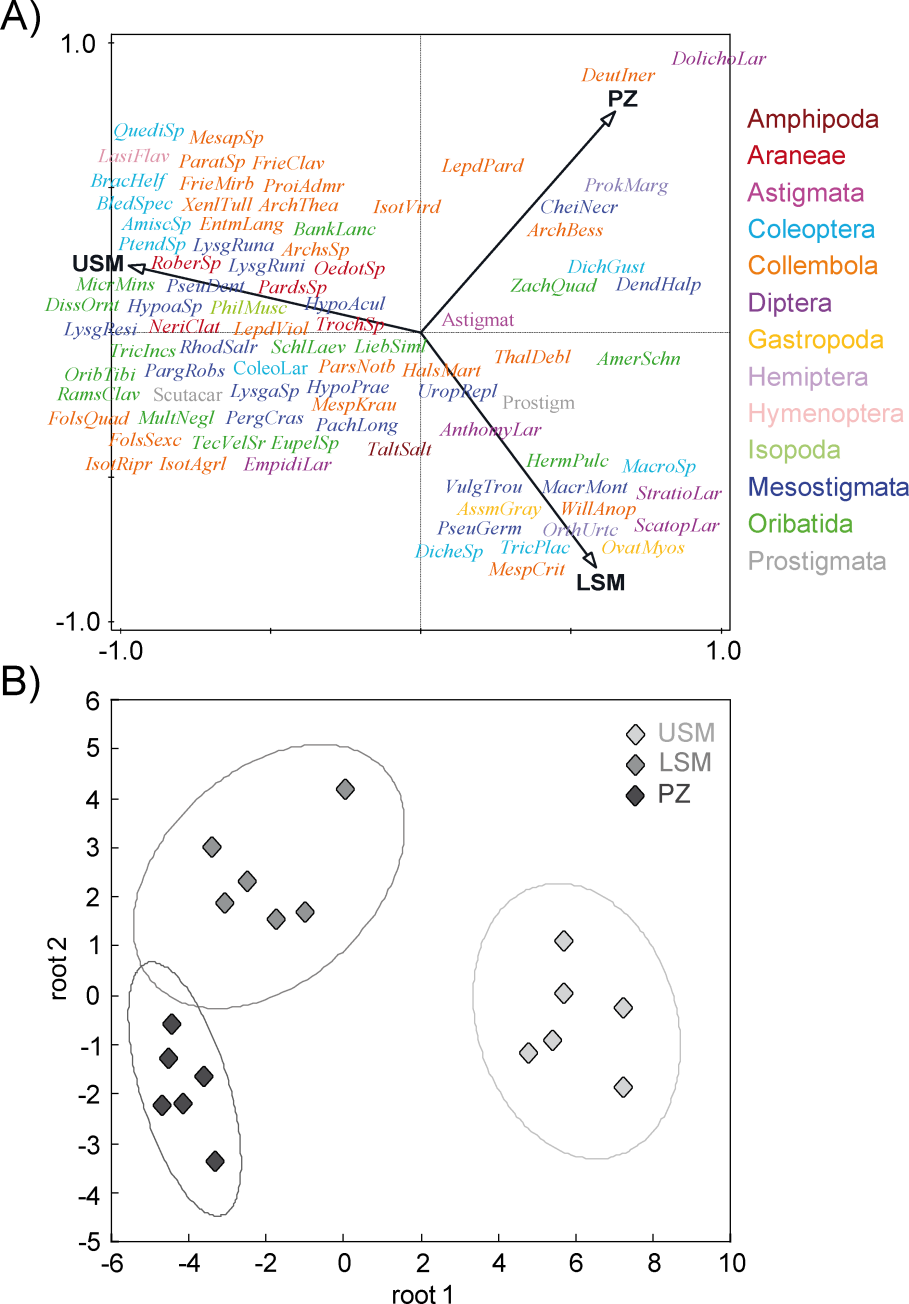
(A) Soil mesofauna community structure: canonical correspondence analysis (CCA) based on the density of 86 soil animal taxa (log-transformed) along the studied salt marsh gradient: upper salt marsh (USM), lower salt marsh (LSM) and pioneer zones (PZ). For full species names see Table A in S1 File. Eigenvalues of axis 1 = 0.6610 and axis 2 = 0.3214. (B) Discriminant function analysis (DFA) of soil invertebrate community of the three salt marsh zones: USM, LSM and PZ; ellipses represent confidence ranges at p < 0.05.

The salt marsh soil community comprised 86 taxa, of which 61 were identified to species level: 1 Amphipoda, 5 Araneae, 1 Astigmata, 9 Coleoptera, 24 Collembola, 5 Diptera, 2 Gastropoda, 2 Hemiptera, 1 Hymenoptera, 1 Isopoda, 19 Mesostigmata, 14 Oribatida and 2 Prostigmata (see Table A in S1 File for full species list). Canonical correspondence analysis (CCA) separated the community of the upper salt marsh from that of the lower salt marsh and pioneer zone, reflecting the variations in distribution patterns of soil mesofauna between the vegetation zones (Fig 2A). Discriminant function analysis (DFA) indicated that soil animal community structure of the upper salt marsh significantly differenced between and that of the lower salt marsh and the pioneer zone (Fig 2B; Tukey’s HSD: p < 0.0001).

**Fig 3:**
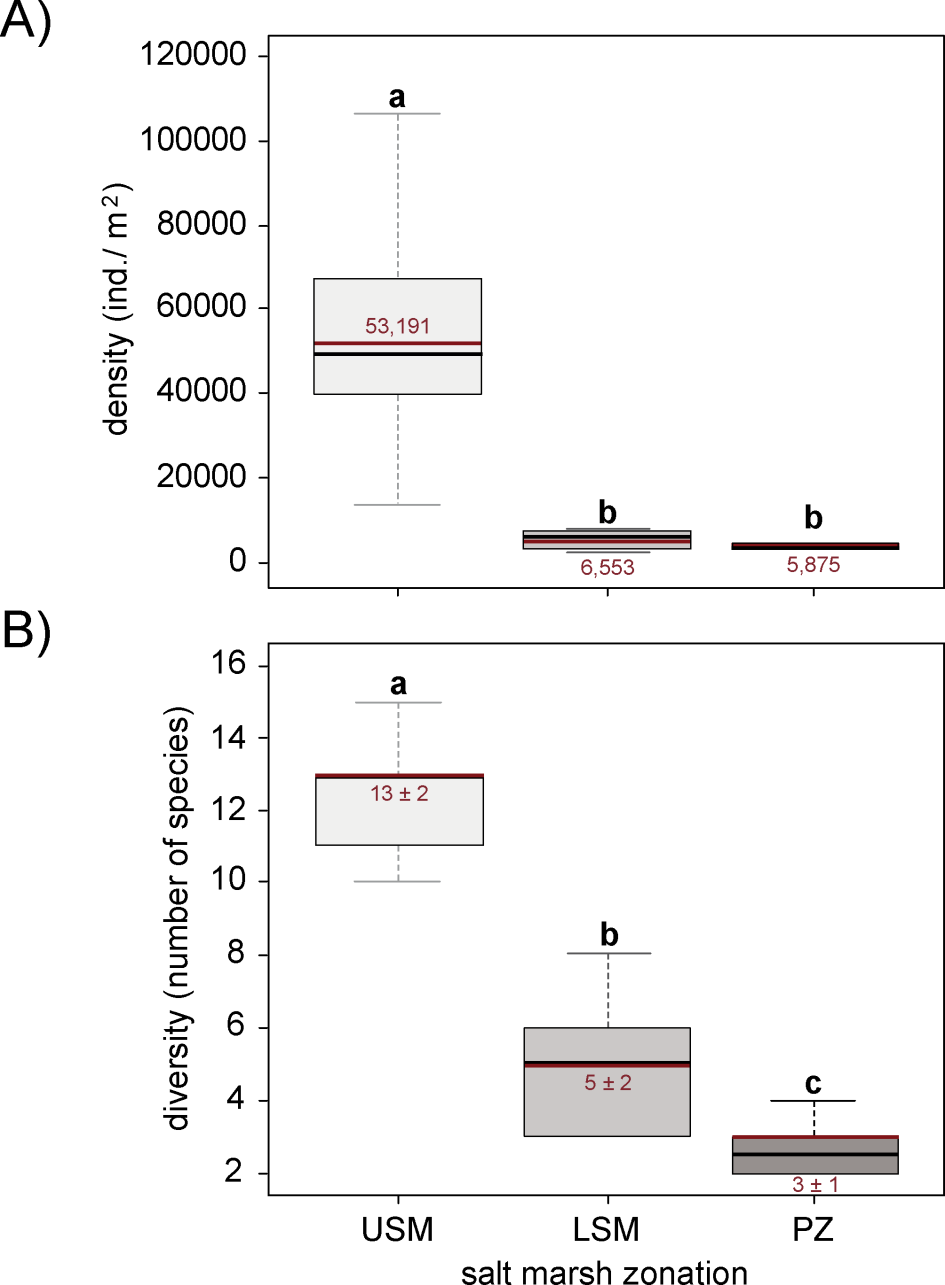
(A) Density and (B) diversity of soil mesofauna species at the upper salt marsh (USM), lower salt marsh (LSM) and pioneer zone (PZ). Boxplots represent mean (red line) and median (black line) of density and diversity. Different letters represent significant differences between zones (Tukey’s HSD test, p < 0.05).

Density of the soil mesofauna significantly declined from the upper salt marsh to the lower salt marsh and pioneer zone (*F*_2,15_ = 13.26, p < 0.001; Fig 3A). Similarly, species diversity varied significantly between the three salt marsh zones (*F*_2,15_ = 61.18, p < 0.001; Fig 3B), but declined more continuously from the upper salt marsh to the lower salt marsh to the pioneer zone. The mesofauna was dominated by species of Collembola, Oribatida and Mesostigmata, which mainly occurred in the upper and lower salt marsh (see Table A in S1 File for species list). In contrast, the pioneer zone was dominated by species connected to littoral habitats with high frequency of inundation, e.g. the two Collembola species *Archisotoma besselsi* and *Thalassaphorura debilis*, the three Oribatida species *Ameronothrus schneideri*, *Hermannia pulchella* and *Zachvatkinibates quadrivertex*, and the Mesostigmata species *Cheiroseius necorniger* and *Dendrolaelaps halophilus*.

### Trophic structure

**Fig 4:**
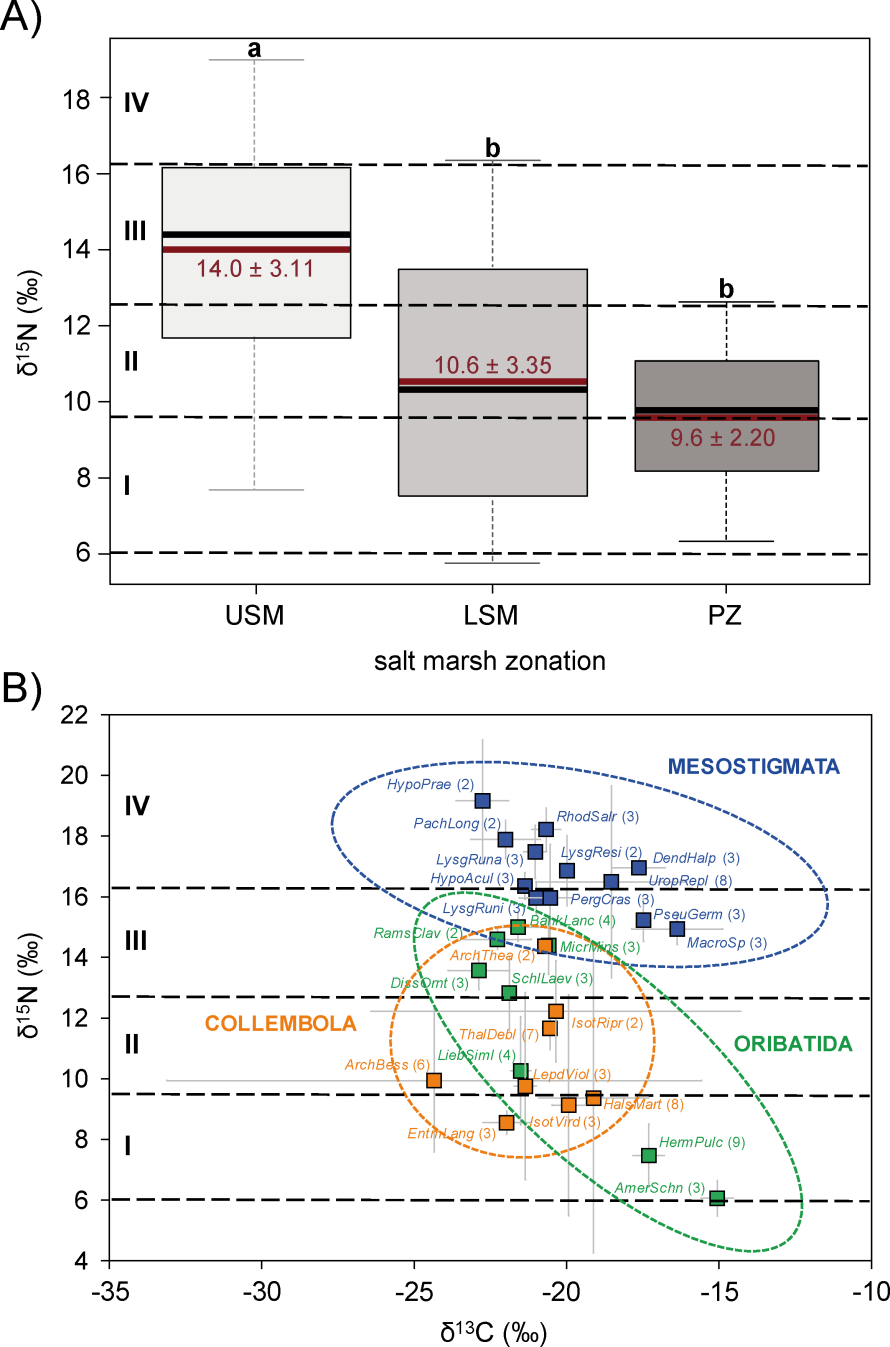
(A) δ^15^N signatures of all soil mesofauna species in the upper salt marsh (USM), lower salt marsh (LSM) and pioneer zone (PZ). Boxplots represent mean (red line) and median (black line) of δ^15^N signatures; different letters represent significant differences between zones (Tukey’s HSD; p < 0.05). (B) δ^13^C and δ^15^N stable isotope values (means with standard deviation) of dominant mesofauna species: Collembola (orange squares), Mesostigmata (blue squares) and Oribatida (green squares). Black dashed horizontal lines represent estimated trophic level boundaries with each trophic level spanning 3.4‰ δ^15^N: I = primary decomposers, II = secondary decomposers, III = first order predators, and IV = second order predators. Number of replicates are included in parentheses; see Table C in S1 File for full species names.

As indicated by δ^15^N signatures, the complete mesofauna food web consisted of four trophic levels: (I) primary decomposers (6.1–9.5‰), (II) secondary decomposers (9.5–12.9‰), (III) first order predators (12.9–16.3‰) and (IV) second order predators (16.3–19.7‰). However, the trophic structure of the mesofauna food web changed significantly along the land sea transect (F_2,50_ = 9.08, p < 0.000). On average, δ^15^N values of the upper salt marsh (14.4 ± 3.14‰) exceeded those of the lower salt marsh (11.4 ± 4.23‰) and pioneer zone (9.1 ± 3.24‰) suggesting shorter food chains in the latter two zones, with the food web in the upper salt marsh comprising four trophic levels whereas that in the pioneer zone comprising only two trophic levels (Fig 4A, Table B in S1 File).

δ^15^N signatures of mesofauna species spanned from 6.1 ± 0.72‰ in *A*. *schneideri* (Oribatida) to 19.2 ± 2.01‰ in *Hypoaspis praesternalis* (Mesostigmata) reflecting the four trophic levels the upper salt marsh (Fig 4B, Table C in S1 File). In general, the number of primary decomposers, i.e., animals that feed mainly on organic matter, was low. Only the Oribatida species *A*. *schneideri* had ^15^N signatures close to that of organic matter. Collembola species were positioned in trophic levels I to III with δ^15^N values ranging between 8.6 ± 0.38‰ and 14.4 ± 0.07‰ (see Table C in S1 File). Most of the Collembola species were ascribed to primary or secondary decomposers with the exception of *Archisotoma theae*, which was positioned in trophic level III indicating first order predator lifestyle (Fig 4B). Similarly, Oribatida spanned from trophic level I to III with δ^15^N values ranging between 6.1 ± 0.72 and 15.0 ± 0.46‰ (see Table C in S1 File). Contrasting Collembola, however, most Oribatida species (*Banksinoma lanceolata*, *Dissorhina ornata*, *Microppia minus*, *Ramusella clavipectinata* and *Scheloribates laevigatus*) were ascribed to trophic level III, i.e. first order predators (Fig 4B). As expected, Mesostigmata species had the highest δ^15^N values ranging from 14.9 ± 0.50 to 19.2 ± 2.01‰ (see Table C in S1 File). Accordingly, the Mesostigmata community was composed of first and second order predators, i.e. trophic level III and IV (Fig 4B). High δ^15^N signatures of Mesostigmata species indicate that beside secondary decomposers, first order predators such as certain Oribatida and Collembola species as well as other Mesostigmata species significantly contribute to their diet.

### Linkage of basal resources to consumers

**Fig 5:**
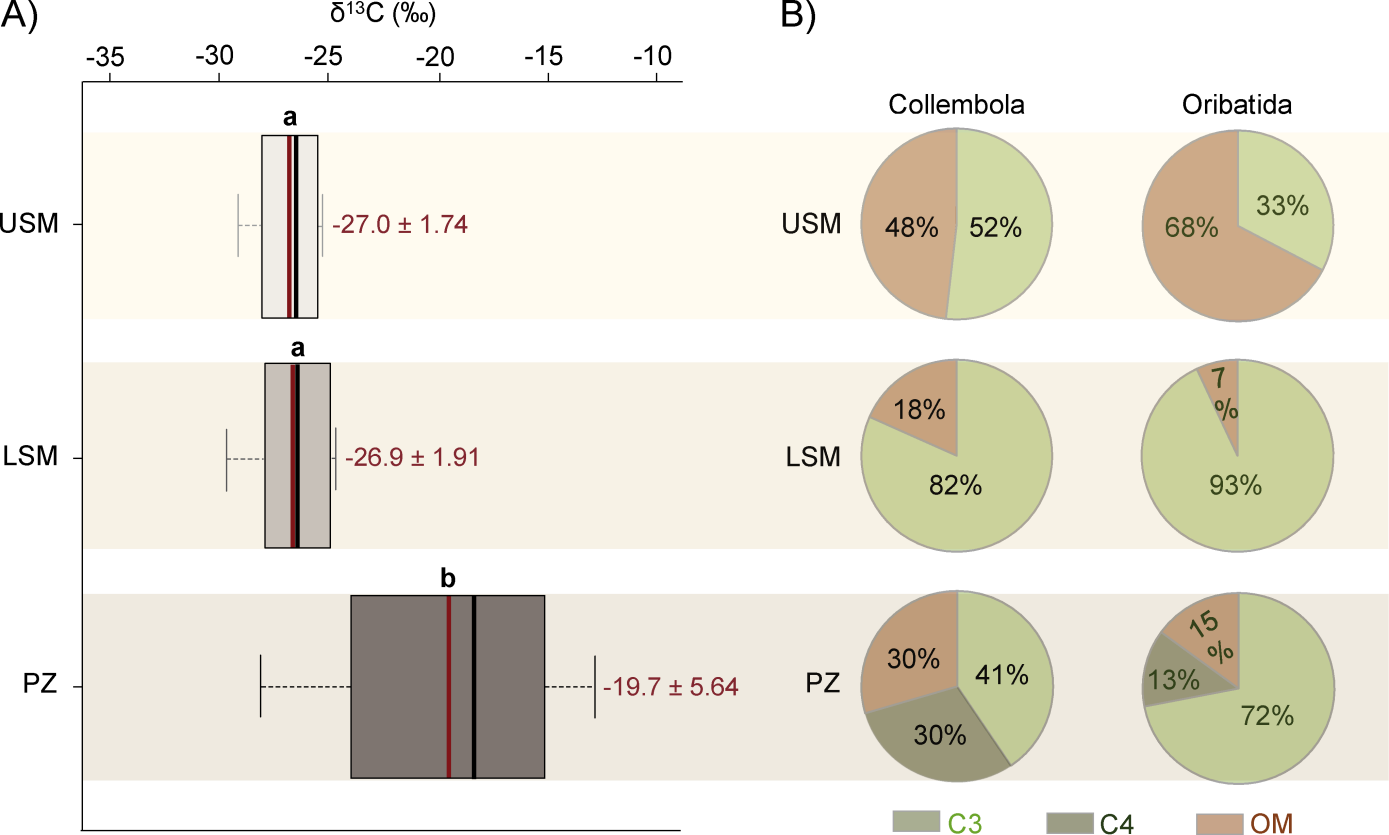
(A) δ^13^C signatures of basal food resources in the three salt marsh zones: upper salt marsh (USM), lower salt marsh (LSM) and pioneer zones (PZ). Boxplots represent mean (red line) and median (black line) of δ^13^C signatures; different letters represent significant differences between vegetation zones (Tukey’s HSD, p < 0.05). (B) Pie charts represent mean percentages of potential food resources [C3 plants/algae, C4 plants/algae and organic material (OM) containing C3 and/or C4 plants/algae] of Collembola and Oribatida species along the salt marsh gradient.

The composition of basal food resources changed significantly along the land-sea vegetation zonation (*F*_1,18_ = 7.25, p = 0.0049). δ^13^C values of basal food resources of the pioneer zone differed significantly from those of the upper and lower salt marsh (Fig 5A). δ^13^C values of basal food resources increased from -27.0 ± 1.7‰ and -26.9 ± 1.9‰ in the upper and lower salt marsh, respectively, to -19.7 ± 5.6‰ in the pioneer zone (Table D in S1 File).

Mixing models suggested that Collembola and Oribatida species prefer different resources within the salt marsh zones. The upper and lower salt marsh was dominated by C3 plants and soil organic matter (containing C3 plant carbon), whereas the pioneer zone was characterized by C3 and C4 plants/algae and soil organic matter containing C3 and C4 plants/algae carbon (Fig A and Table D in S1 File). In the upper salt marsh, Collembola predominantly rely on C3 plants with 52% of their body carbon originating from this source, whereas Oribatida rely on soil organic matter with 68% of their body carbon originating from this source (Fig 5B, Table E in S1 File). In contrast, resource use of Collembola and Oribatida in the lower salt marsh strongly shifted towards C3 plants with 82% and 93% of their body carbon originating from this source and only 18% of Collembola and 7% of Oribatida body carbon originating from soil organic matter (Fig 5B, Table E in S1 File). In the pioneer zone, Collembola predominantly rely on C3 plants with a contribution of 41% and in addition use C4 plants/algae as well as organic material to a similar extent, i.e. 30% each. In comparison, Oribatida species predominantly rely on C3 plants with 72% of their body carbon originating from this source and only 13% and 15% originating from C4 plants/algae and organic material, respectively.

As indicated by the Collembola species *T*. *debilis*, which inhabited all three salt marsh zones (see Table A in S1 File), resource use of species changed along the salt marsh gradient. Based on mixing models this species predominantly incorporated C3 plant carbon in the upper and lower salt marsh (74% and 90%, respectively) whereas in the pioneer zone it predominantly fed on marine C4 plants/algae (37%; Table E in S1 File).

## Discussion

### Disturbance and community structure

Density and diversity of soil mesofauna decreased significantly with increasing levels of inundation frequency from the land towards the sea. This indicates that the observed community shift correlated with changes in flooding from the upper salt marsh to the pioneer zone [22, 23, 65]. An increasing level of inundation frequency to the sea induces increasing stress for mesofauna species due to variable but generally high salinity (salinity of 5–20, 20–26 and 26–32 for the upper salt marsh, lower salt marsh and pioneer zone, respectively; D. Meier, pers. comm.).

The upper salt marsh is less disturbed by inundations as those are restricted to occasional storm events, therefore species with low salt tolerance can thrive in this habitat. Accordingly, the soil fauna was dominated by species typical for terrestrial meadows i.e., *Entomobrya lanuginosa*, species of *Folsomia*, *Isotoma* and *Lepidocyrtus* (Collembola), *Liebstadia similis*, *Multioppia neglecta*, *R*. *clavipectinata*, *S*. *laevigatus* and *Tectocepheus velatus sarekensis* (Oribatida), *Rhodacarus salaries* and *Uropoda repleta* (Mesostigmata). Abiotic disturbances become more frequent in the lower salt marsh and in particular in the pioneer zone. Therefore, the lower salt marsh was inhabited by salt and desiccation tolerant species, such as *Halisotoma maritima* and *Mesaphorura krausbaueri* (Collembola), *H*. *pulchella* (Oribatida), and *Pseudoparasitus germanicus* (Mesostigmata). The pioneer zone is the most extreme habitat of the salt marsh with highest frequency of inundations and abiotic variations. Therefore, specialist species preferentially colonize this salty marine habitat, such as *A*. *besselsi* and *T*. *debilis* (Collembola), *A*. *schneideri*, *H*. *pulchella* and *Z*. *quadrivertex* (Oribatida), and *C*. *necorniger* and *D*. *halophilus* (Mesostigmata). Colonization of the lower salt marsh and pioneer zone is associated with physiological and behavioral adaptations enabling the species to cope with frequent inundations and variations in salinity.

Certain Collembola species are osmoconform even at high salinity, whereas others are drifting on the seawater surface allowing aerial respiration [66–68], whereas non-haloric Oribatida species developed plastron structures allowing them to respire under water [69]. Halobiont Oribatida, such as *A*. *schneideri*, *H*. *pulchella* and *Z*. *quadrivertex*, are well adapted to saline conditions and are able to withstand frequent inundation and to survive in submersion [38, 46, 70, 71]. In contrast, nothing is known about the mechanisms which enable Mesostigmata, such as the *C*. *necorniger* and *D*. *halophilus*, to inhabit frequently inundated salt marsh zones. However, widespread occurrence in the lower salt marsh and the pioneer zone suggests that they are well adapted to harsh environmental conditions and frequent inundation.

Declining diversity of mesofauna in salt marsh zones closer to the sea is in line with the “Intermediate Disturbance Hypothesis” stating that species diversity is maximized when ecological disturbance is neither too rare nor too frequent [72]. Terrestrial species not well adapted to abiotic disturbances are likely to be excluded from the semi-marine lower salt marsh zones resulting in lower species density and diversity. As a consequence, specialized taxa are most affected by disturbances, but able to tolerate the abiotic fluctuations and survive under extreme environmental conditions close to the sea due to physiological and behavioral adaptations [73, 74].

### Disturbance and food web structure

Salt marsh soils provide a wide range of food resources for soil fauna communities. The δ^15^N signatures suggest that mesofauna species of salt marshes occupy very different trophic niches. The food web spans over four trophic levels including primary and secondary decomposers, as well as first and second order predators, suggesting that intra-guild predation is widespread and plays an important role. The broad range of δ^13^C signatures of salt marsh mesofauna species supports the hypothesis, that the utilization of diverse food resources along the salt marsh gradient fundamentally structures the entire food web.

Supporting the predominant role of disturbance in structuring the soil mesofauna food web, the number of trophic levels was higher in the less disturbed upper salt marsh zone and decreased to the pioneer zone. Notably, the structure of the food web at the higher salt marsh zones closely resembled that of forest ecosystems characterized by more opportunistic links and long food chains, which typically span over four trophic levels [75, 76]. Previous studies in forest ecosystems also showed Oribatida to span three to four trophic levels including primary and secondary decomposers as well as predators and/or scavengers [77]. Similar results have been obtained for Collembola with again most species functioning as secondary rather than primary decomposers [78, 79]. This suggests that similar to forest ecosystems, most decomposer taxa of salt marsh habitats do not feed on dead organic matter but occupy higher trophic levels including secondary decomposers (feeding predominantly on microorganisms), predators and/or scavengers. Presumably, similar to forests and arable systems [80, 81], rhizosphere microorganisms relying on root derived resources play an important role as food resource for decomposer species in higher salt marsh zones. In contrast, in the pioneer zone bacteria relying on marine detritus as well as algae play a major role as food resource for Collembola and Oribatida. Supporting this view the generalist Collembola species *T*. *debilis* predominantly fed on marine C4 plants/algae in the pioneer zone but predominantly on C3 plant carbon in the upper salt marsh. Similarly, other studies showed that the Collembola species *Archisotoma pulchella* prefers to feed on diatoms [82] and the Oribatida species *Ameronothrus lineatus* predominantly feeds on green algae [83, 84]. Previous studies suggested that marine resources provide most energy and resources for coastal communities and their importance extends considerably into the terrestrial realm [5]. In contrast to these findings, the use of marine-based food sources by soil invertebrates was limited to the pioneer zone, whereas invertebrates at higher salt marsh zones predominantly relied on terrestrial resources of these habitats. This points to the importance of marine resources for soil food webs of frequently inundated salt marsh habitats, whereas higher salt marsh zones, colonized by terrestrial plants, provide most of the resources for the mesofauna decomposer system. On the other hand, the results indicate that soil mesofauna species do not migrate between the salt marsh zones thereby integrating resources of different habitats.

Further, the results suggest that soil mesofauna food webs of salt marshes predominantly dwell on autochthonous resources based in large on the plants (including algae) colonizing the respective salt marsh zones. In the upper and lower salt marsh root derived resources are likely to play an important role in fueling the decomposer system, thereby resembling truly terrestrial systems such as forests but also arable systems [80, 81]. Consequently, the shift from a typical terrestrial plant system in the higher salt marsh zones to a more marine plant system in the pioneer zone is associated with fundamental changes in the decomposer system, which in turn affects the food web structure with a decreasing number of trophic levels to the sea (pioneer zone).

As indicated by high δ^15^N values a number of Collembola and Oribatida species function as predators and/or scavengers. Predator Collembola and Oribatida species likely are feeding at least in part on nematodes, which are highly abundant in salt marshes, similar to terrestrial soil food webs [85–87]. Notably, the results suggest predation to be more widespread in Oribatida than in Collembola. Conform to our expectations and previous studies of forest soil food webs [42], however, Mesostigmata formed the main predators in the studied salt marsh mesofauna food webs. Their high δ^15^N values and variable hunting strategies [88] suggest complex predator-prey relationships including intraguild predation. Further, Mesostigmata themselves likely function as prey of marine predators such as nematodes, copepods and polychaetes and this potentially contributed to the lower number of trophic levels of the mesofauna food web in the pioneer zone. By contrast, reduced predation by marine species may have contributed to the higher abundance and diversity of mesofauna, and the longer food chains in the lower and upper salt marsh [43]. However, in these habitats mesofauna likely forms important prey of macrofauna predators such as Araneae. Therefore, the relative contribution of predation as structuring force of mesofauna communities in the different salt marsh zones remains uncertain and needs further investigation.

A large number of mesofauna species (60 taxa) only occurred in one of the three vegetation zones, suggesting that habitat and / or trophic specialists are widespread. Trophic specialists occupy a limited range of niches but more effectively exploit these resources than trophic generalists [27, 89]. In contrast, 22 taxa colonized two salt marsh zones underlining that salt marsh communities are well structured with most species only being able to colonize a narrow habitat range and use the respective resources of that habitat. Only two Collembola species (*A*. *besselsi* and *T*. *debilis*) typically colonizing disturbed habitats, occurred in each of the three salt marsh zones and therefore truly represent generalists. Food generalists opportunistically use resources which are available [90] allowing them to colonize a wide range of habitats [6, 91]. However, even though habitat specialists predominated, these species presumably also used a wider range of resources rather than only single prey taxa as reflected by the dominance of secondary decomposers as well as second order predators indicating that the use of resources of different trophic levels [92] is widespread among salt marsh mesofauna species. Presumably, feeding on multiple trophic levels is favored in the heterogeneous salt marsh habitats and this likely contributes to food web stability [93].

Body mass is a major structuring factor of food webs [94], especially predator-prey interactions strongly depend on body mass ratios, therefore we expected large species to predominante in higher trophic levels. Contrasting this expectation and other food webs [95], the trophic position of mesofauna species was not significantly related to body mass (Fig B and Table B in S1 File). Again, however, this resembles terrestrial Mesostigmata communities of forests in Central Europe [42].

## Conclusions

The study highlighted the complex trophic structure of mesofauna communities and their central position within salt marsh habitats. Strong changes in community structure and food web complexity along the studied salt marsh gradient suggest that the occurrence of the majority of species in salt marshes is related to inundation frequency. Parallel to the strong turnover of species, mesofauna food webs markedly changed along the salt marsh gradient from four trophic levels in the upper salt marsh to only two in the pioneer zone. Similar to terrestrial soil food webs, the number of primary decomposers was low and most species functioned as secondary decomposers or predators including second order predators or scavengers. In particular the high number of second order predator taxa suggests high incidence of intraguild predation, again resembling terrestrial soil food webs. Notably, soil animal communities in each of the three salt marsh zones predominantly relied on autochthonous resources, with marine resources being restricted mainly to the pioneer zone, and indicating that terrestrial food webs are intimately linked to rhizosphere resources. Overall, the high number of species of soil microarthropods including Collembola, Oribatida and Mesostigmata suggests that, similar to consolidated terrestrial systems, these taxa fill key trophic positions of soil food webs even at the adverse environmental conditions of marine–terrestrial boundary habitats.

## Supporting information

S1 File **Figure A.** Mean δ^13^C values ± standard deviation (SD) of basal food resources (vascular plants, algae and soil organic matter) of the three salt marsh zones: upper salt marsh (USM), lower salt marsh (LSM) and pioneer zones (PZ). Light grey squares mark C3 plants/algae, black squares mark C4 plants/algae. **Figure B.** Relationship between body mass and trophic level as indicated by δ^15^N signatures of Mesostigmata (blue), Collembola (orange) and Oribatida species (green) for the three salt marsh zones: upper salt marsh (USM, dots), lower salt marsh (LSM, 296 triangles) and pioneer zone (PZ, diamonds). Dry weight of species is given in Table B in S1 File. **Table A.** List of soil invertebrate taxa and abbreviations. Mean density (ind./m^2^) and standard deviation (SD) of mesofauna species in the three salt marsh zones: upper salt marsh, lower salt marsh and pioneer zone. **Table B.** Mean stable isotope ratios of δ^15^N values (‰) and standard deviation (SD) of soil invertebrate taxa of the three salt marsh zones: upper salt marsh, lower salt marsh and pioneer zone. Table including number of replicates (*n*) and dry weight of individuals. **Table C.** Trophic structure–mean stable isotope ratios of δ^13^C and δ^15^N values (‰) and standard deviation (SD) of dominant species of Collembola, Mesostigmata and Oribatida, including taxa abbreviations and number of replicates (*n*). Stable isotope signatures were normalized to the mean of the organic matter signature of the respective salt marsh zone. **Table D.** Mean δ^13^C (‰) and standard deviation (SD) of basal food resources of the three salt marsh zones: upper salt marsh, lower salt marsh and pioneer zone, table including sample size (*n*) and type of CO_2_-fixation. **Table E.** Mixing models–mean percentage of potential food sources [C3 plants/algae, C4 plants/algae and organic material (OM) containing C3 and/or C4 plants/algae] calculated by mean values and standard deviation (SD) of stable isotope signatures (δ^13^C and δ^15^N) of Collembola and Oribatdia consumer species in the upper salt marsh, lower salt marsh and pioneer zone.
(DOC)
