# Supplementary material for: The structure of salt marsh soil mesofauna food webs – The prevalence of disturbance
Source: PLoS One. 2017 Dec 14;12(12):e0189645. doi: 10.1371/journal.pone.0189645 (PMC5730192; doi:10.1371/journal.pone.0189645)
Supplement: S1 File — Figure A. Mean δ13C values ± standard deviation (SD) of basal food resources (vascular plants, algae and soil organic matter) of the three salt marsh zones: upper salt marsh (USM), lower salt marsh (LSM) and pioneer zones (PZ). Light grey squares mark C3 plants/algae, black squares mark C4 plants/algae. Figure B. Relationship between body mass and trophic level as indicated by δ15N signatures of Mesostigmata (blue), Collembola (orange) and Oribatida species (green) for the three salt marsh zones: upper salt marsh (USM, dots), lower salt marsh (LSM, 296 triangles) and pioneer zone (PZ, diamonds). Dry weight of species is given in Table B in S1 File. Table A. List of soil invertebrate taxa and abbreviations. Mean density (ind./m2) and standard deviation (SD) of mesofauna species in the three salt marsh zones: upper salt marsh, lower salt marsh and pioneer zone. Table B. Mean stable isotope ratios of δ15N values (‰) and standard deviation (SD) of soil invertebrate taxa of the three salt marsh zones: upper salt marsh, lower salt marsh and pioneer zone. Table including number of replicates (n) and dry weight of individuals. Table C. Trophic structure–mean stable isotope ratios of δ13C and δ15N values (‰) and standard deviation (SD) of dominant species of Collembola, Mesostigmata and Oribatida, including taxa abbreviations and number of replicates (n). Stable isotope signatures were normalized to the mean of the organic matter signature of the respective salt marsh zone. Table D. Mean δ13C (‰) and standard deviation (SD) of basal food resources of the three salt marsh zones: upper salt marsh, lower salt marsh and pioneer zone, table including sample size (n) and type of CO2-fixation. Table E. Mixing models–mean percentage of potential food sources [C3 plants/algae, C4 plants/algae and organic material (OM) containing C3 and/or C4 plants/algae] calculated by mean values and standard deviation (SD) of stable isotope signatures (δ13C and δ15N) of Collembola and Oribatdia con [file pone.0189645.s001.doc]

**Figure A**

**
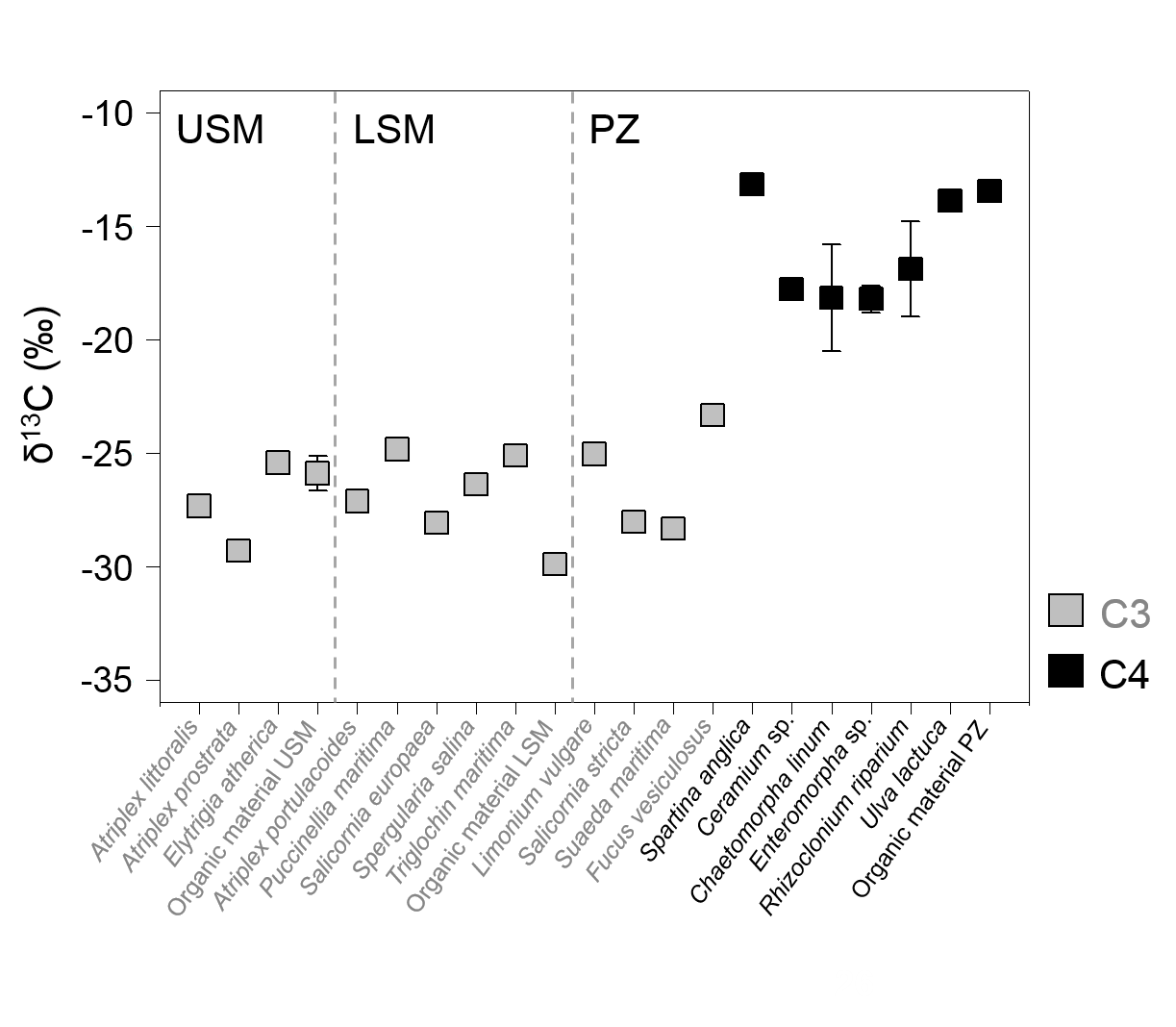
**

**Figure B**

**
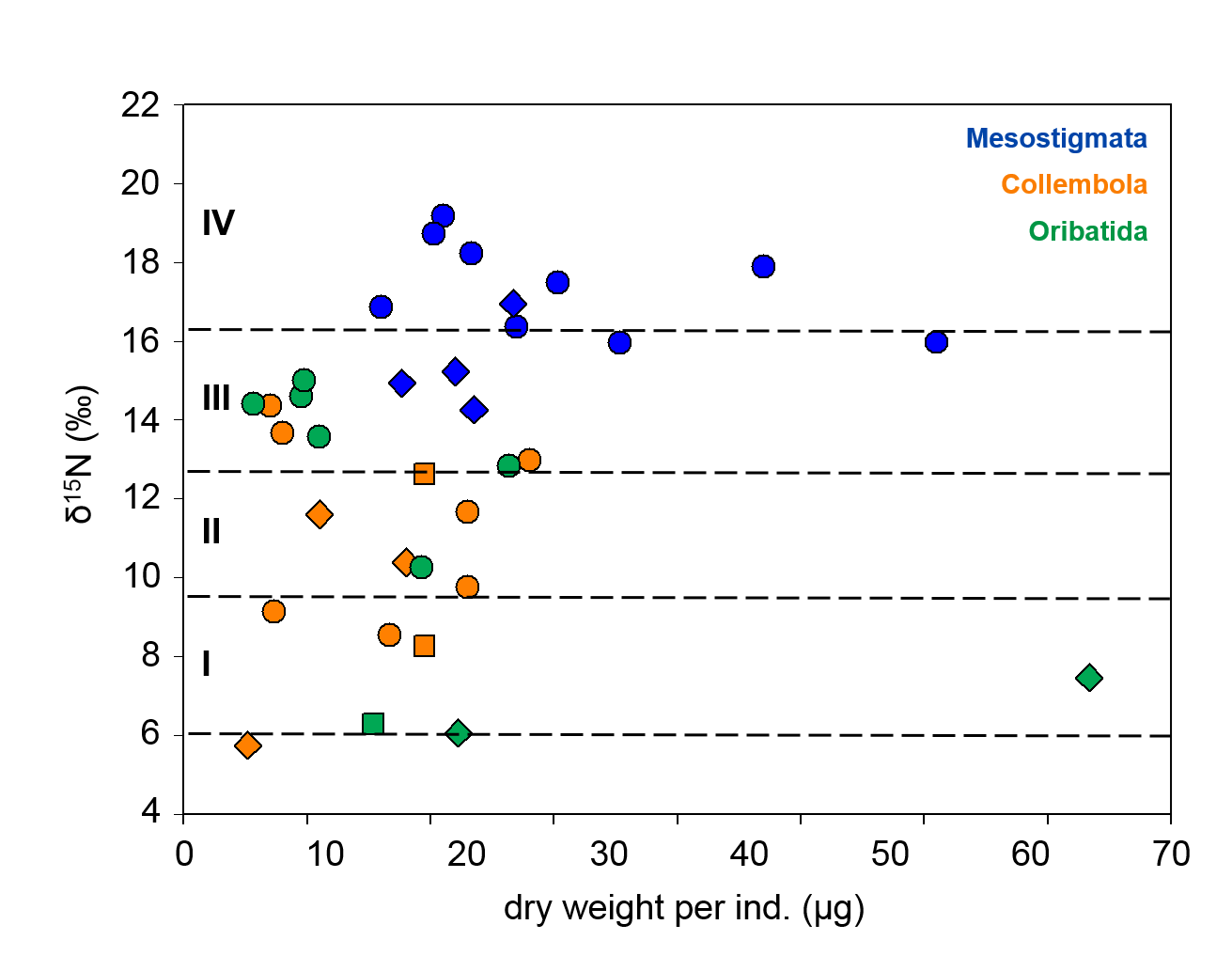
**

**Table A**

| **soil invertebrate taxa** | | **abbreviation** | **density (ind./ m^2^)** | | | | | |
| --- | --- | --- | --- | --- | --- | --- | --- | --- |
|  |  |  | **upper salt marsh** | | **lower salt marsh** | | **pioneer zone** | |
|  |  |  | **mean** | **SD** | **mean** | **SD** | **mean** | **SD** |
| Amphipoda | |  |  |  |  |  |  |  |
|  | *Talitrus saltator* | *TaltSalt* | 732 | 160.12 | 764 | 359.92 |  |  |
| Araneae | |  |  |  |  |  |  |  |
|  | *Neriene clathrata* | *NeriClat* | 509 | 0.00 |  |  |  |  |
|  | *Oedothorax* sp. | *OedotSp* | 509 | 0.00 |  |  |  |  |
|  | *Pardosa* sp. | *PardsSp* | 509 | 0.00 |  |  |  |  |
|  | *Robertus* sp. | *RoberSp* | 1018 | 719.83 |  |  |  |  |
|  | *Trochosa* sp. | *TrochSp* | 764 | 359.92 |  |  |  |  |
| Astigmata | | Astigmat | 1209 | 759.96 | 1018 | 719.83 | 509.00 | 0.00 |
| Coleoptera | |  |  |  |  |  |  |  |
|  | *Amischa* sp. | *AmiscSp* | 700 | 269.94 |  |  |  |  |
|  | *Bledius spectabilis* | *BledSpec* | 509 | 0.00 |  |  |  |  |
|  | *Brachygluta helferi* | *BracHelf* | 509 | 0.00 |  |  |  |  |
|  | *Dicheirotrichus gustavii* | *DichGust* |  |  | 509 | 0.00 | 509 | 0.00 |
|  | *Dicheirotrichus* sp. | *DicheSp* |  |  | 509 | 0.00 |  |  |
|  | *Ptenidium* sp. | *PtendSp* | 1273 | 1322.42 |  |  |  |  |
|  | *Quedius* sp. | *QuediSp* | 509 | 0.00 |  |  |  |  |
|  | *Trichocellus placidus* | *TricPlac* |  |  | 509 | 0.00 |  |  |
|  | Coleoptera larvae | ColeoLar | 573 | 127.25 | 1018 | 0.00 |  |  |
| Collembola | |  |  |  |  |  |  |  |
|  | *Archisotoma besselsi* | *ArchBess* | 1451 | 1509.94 | 859 | 543.61 | 4348 | 3737.27 |
|  | *Archisotoma theae* | *ArchThea* | 22396 | 0.00 |  |  |  |  |
|  | *Archisotoma* sp | ArchsSp | 509 | 0.00 |  |  |  |  |
|  | *Deuteraphorura inermis* | *DeutIner* |  |  |  |  | 509 | 0.00 |
|  | *Entomobrya lanuginosa* | *EntmLang* | 679 | 308.21 |  |  |  |  |
|  | *Folsomia quadrioculata* | *FolsQuad* | 1527 | 0.00 |  |  |  |  |
|  | *Folsomia sexoculata* | *FolsSexc* | 933 | 529.78 |  |  |  |  |
|  | *Friesea claviseta* | *FrieClav* | 795 | 334.66 |  |  |  |  |
|  | *Friesea mirabilis* | *FrieMirb* | 509 | 0.00 |  |  |  |  |
|  | *Halisotoma maritima* | *HalsMart* | 12343 | 12437.34 | 891 | 661.21 |  |  |
|  | *Isotoma agrelli* | *IsotAgrl* | 509 | 0.00 |  |  |  |  |
|  | *Isotoma riparia* | *IsotRipr* | 848 | 293.87 |  |  |  |  |
|  | *Isotoma viridis* | *IsotVird* | 1103 | 388.76 |  |  | 509 | 0.00 |
|  | *Lepidocyrtus paradoxus* | *LepdPard* | 509 | 0.00 |  |  | 509 | 0.00 |
|  | *Lepidocyrtus violaceus* | *LepdViol* | 2460 | 2149.99 | 509 | 0.00 |  |  |
|  | *Mesaphorura critica* | *MespCrit* |  |  | 764 | 0.00 |  |  |
|  | *Mesaphorura krausbaueri* | *MespKrau* | 5111 | 4794.08 | 814 | 331.83 |  |  |
|  | *Mesaphorura* sp. | MesapSp | 509 | 0.00 |  |  |  |  |
|  | *Paratullbergia* sp. | *ParatSp* | 509 | 0.00 |  |  |  |  |
|  | *Parisotoma notabilis* | *ParsNotb* | 891 | 328.56 | 1527 | 0.00 |  |  |
|  | *Proisotoma admaritima* | *ProiAdmr* | 1782 | 0.00 |  |  |  |  |
|  | *Thalassaphorura debilis* | *ThalDebl* | 3309 | 3599.17 | 976 | 446.89 | 2545 | 0.00 |
|  | *Willemia anophtalma* | *WillAnop* |  |  | 509 | 0.00 |  |  |
|  | *Xenylla tullbergi* | *XenlTull* | 509 | 0.00 |  |  |  |  |
| Diptera | |  |  |  |  |  |  |  |
|  | Anthomyiidae larvae | AnthomyLar | 509 | 0.00 | 1018 | 0.00 |  |  |
|  | Dolichopodidae larvae | DolichoLar |  |  |  |  | 509 | 0.00 |
|  | Empididae larvae | EmpidiLar | 509 | 0.00 |  |  |  |  |
|  | Scatopsidae larvae | ScatopLar |  |  | 1273 | 440.81 |  |  |
|  | Stratiomyidae larvae | StratioLar |  |  | 764 | 359.92 |  |  |
| Gastropoda | |  |  |  |  |  |  |  |
|  | *Assiminea grayana* | *AssmGray* |  |  | 1188 | 587.74 |  |  |
|  | *Ovatella myosotis* | *OvatMyos* |  |  | 509 | 0.00 |  |  |
| Hemiptera | |  |  |  |  |  |  |  |
|  | *Orthezia urticae* | *OrthUrtc* |  |  | 509 | 0.00 |  |  |
|  | *Prokelisia marginata* | *ProkMarg* |  |  | 509 | 0.00 | 912 | 548.31 |
| Hymenoptera | |  |  |  |  |  |  |  |
|  | *Lasius flavus* | *LasiFlav* | 1527 | 0.00 |  |  |  |  |
| Isopoda | |  |  |  |  |  |  |  |
|  | *Philoscia muscorum* | *PhilMusc* | 1298 | 571.92 |  |  |  |  |
| Mesostigmata | |  |  |  |  |  |  |  |
|  | *Cheiroseius necorniger* | *CheiNecr* |  |  | 509 | 0.00 | 712.60 | 278.79 |
|  | *Dendrolaelaps halophilus* | *DendHalp* |  |  | 1680 | 1389.30 | 1425.20 | 619.49 |
|  | *Hypoaspis aculeifer* | *HypoAcul* | 509 | 0.00 |  |  |  |  |
|  | *Hypoaspis praesternalis* | *HypoPrae* | 764 | 359.92 | 509 | 0.00 |  |  |
|  | *Hypoaspis* sp. (juvenile) | *HypoaSp* | 509 | 0.00 |  |  |  |  |
|  | *Lysigamasus* cf. r*esinae* | *LysgResi* | 509 | 0.00 |  |  |  |  |
|  | *Lysigamasus runcatellus* | *LysgRuna* | 700 | 243.67 |  |  |  |  |
|  | *Lysigamasus runciger* | *LysgRuni* | 662 | 227.63 |  |  |  |  |
|  | *Lysigamasus* sp. (juvenile) | *LysgaSp* | 764 | 254.50 |  |  |  |  |
|  | *Macrochetes montanus* | *MacrMont* |  |  | 509 | 0.00 |  |  |
|  | *Macrochetes* sp. (juvenile) | *MacroSp* |  |  | 636 | 179.96 |  |  |
|  | *Pachylaelaps longicrinitus* | *PachLong* | 509 | 0.00 | 509 | 0.00 |  |  |
|  | *Paragamasus robustus* | *PargRobs* | 509 | 0.00 |  |  |  |  |
|  | *Pergamasus crassipes* | *PergCras* | 509 | 0.00 |  |  |  |  |
|  | *Rhodacarus salarius* | *RhodSalr* | 1323 | 817.77 | 509 | 0.00 |  |  |
|  | *Pseudoparasitus dentatus* | *PseuDent* | 764 | 359.92 |  |  |  |  |
|  | *Pseudoparasitus germanicus* | *PseuGerm* |  |  | 2545 | 0.00 |  |  |
|  | *Uropoda repleta* | *UropRepl* | 3096 | 1666.28 | 942 | 212.93 |  |  |
|  | *Vulgarogamasus trouessarti* | *VulgTrou* |  |  | 509 | 0.00 |  |  |
| Oribatida | |  |  |  |  |  |  |  |
|  | *Ameronothrus schneideri* | *AmerSchn* |  |  | 509 | 0.00 | 509 | 0.00 |
|  | *Banksinoma lanceolata* | *BankLanc* | 1425 | 1143.13 |  |  |  |  |
|  | *Dissorhina ornata* | *DissOrnt* | 2121 | 1655.88 |  |  |  |  |
|  | *Eupelops* sp. (juvenile) | EupelSp | 509 | 0.00 |  |  |  |  |
|  | *Hermannia pulchella* | *HermPulc* |  |  | 1858 | 802.79 | 509 | 0.00 |
|  | *Liebstadia similis* | LiebSiml | 2068 | 1101.40 | 764 | 0.00 |  |  |
|  | *Microppia minus* | *MicrMins* | 4199 | 0.00 |  |  |  |  |
|  | *Multioppia neglecta* | *MultNegl* | 4556 | 3316.32 |  |  |  |  |
|  | *Oribatula tibialis* | *OribTibi* | 1018 | 0.00 |  |  |  |  |
|  | *Ramusella clavipectinata* | *RamsClav* | 1909 | 2094.80 |  |  |  |  |
|  | *Scheloribates laevigatus* | *SchlLaev* | 636 | 220.40 | 509 | 0.00 |  |  |
|  | *Tectocepheus velatus sarekensis* | *TecVelSr* | 636 | 179.96 |  |  |  |  |
|  | *Trichoribates incisellus* | *TricIncs* | 509 | 0.00 |  |  |  |  |
|  | *Zachvatkinibates quadrivertex* | *ZachQuad* |  |  | 679 | 293.87 | 594 | 146.94 |
| Prostigmata | | Prostigm | 509 | 0.00 | 509 | 0.00 | 509 | 0.00 |
|  | Scutacaridae | Scutacar | 1357 | 1059.57 |  |  |  |  |

**Table B**

| **salt marsh** | **invertebrate** | | **replicates** | **dry weight/ ind.** | **δ^15^N (‰)** | |
| --- | --- | --- | --- | --- | --- | --- |
| **zone** | **soil taxa** | | ***n*** | **(µg)** | **mean** | **SD** |
| **upper** | Amphipoda | |  |  |  |  |
| **salt marsh** |  | *Talitrus saltator* | 9 | 855 | 11.78 | 0.85 |
|  | Araneae | |  |  |  |  |
|  |  | *Trochosa* sp. | 2 | 654 | 16.19 | 1.23 |
|  | Coleoptera | |  |  |  |  |
|  |  | *Ptenidium* sp. | 8 | 61 | 7.65 | 0.76 |
|  |  | *Amischa* sp. | 4 | 109 | 13.69 | 0.74 |
|  |  | Coleptera larvae | 5 | 20 | 15.50 | 2.97 |
|  | Collembola | |  |  |  |  |
|  |  | *Entomobrya lanuginosa* | 3 | 17 | 8.55 | 0.38 |
|  |  | *Lepidocyrtus violaceus* | 3 | 23 | 9.76 | 3.09 |
|  |  | *Archisotoma theae* | 2 | 7 | 14.36 | 0.07 |
|  |  | *Halisotoma maritima* | 3 | 28 | 12.98 | 1.47 |
|  |  | *Isotoma riparia* | 2 | 23 | 11.66 | 0.70 |
|  |  | *Isotoma viridis* | 3 | 7 | 9.13 | 3.64 |
|  |  | *Thalassaphorura debilis* | 2 | 8 | 13.66 | 1.87 |
|  | Hymenoptera | |  |  |  |  |
|  |  | *Lasius flavus* | 3 | 162 | 15.18 | 0.24 |
|  | Isopoda | |  |  |  |  |
|  |  | *Philoscia muscorum* | 13 | 1149 | 11.15 | 0.95 |
|  | Mesostigmata | |  |  |  |  |
|  |  | *Hypoaspis aculeifer* | 3 | 27 | 16.36 | 0.43 |
|  |  | *Hypoaspis praesternalis* | 2 | 21 | 19.16 | 2.01 |
|  |  | *Pachylaelaps longicrinitus* | 2 | 47 | 17.89 | 0.63 |
|  |  | *Lysigamasus cf resinae* | 2 | 16 | 16.86 | 1.16 |
|  |  | *Lysigamasus runcatellus* | 3 | 30 | 17.48 | 0.88 |
|  |  | *Lysigamasus runciger* | 3 | 35 | 15.96 | 0.53 |
|  |  | *Pergamasus crassipes* | 3 | 61 | 15.96 | 1.76 |
|  |  | *Rhodocarus salarius* | 3 | 23 | 18.22 | 0.71 |
|  |  | *Uropoda repleta* | 4 | 20 | 18.73 | 0.78 |
|  | Oribatida | |  |  |  |  |
|  |  | *Liebstadia similis* | 4 | 19 | 10.26 | 1.78 |
|  |  | *Dissorhina ornata* | 3 | 11 | 13.57 | 0.62 |
|  |  | *Microppia minus* | 3 | 6 | 14.39 | 0.96 |
|  |  | *Ramusella clavipectinata* | 2 | 10 | 14.59 | 0.34 |
|  |  | *Scheloribates laevigatus* | 3 | 26 | 12.82 | 1.21 |
|  |  | *Banksinoma lanceolata* | 4 | 10 | 15.00 | 0.46 |
|  | Prostigmata | |  |  |  |  |
|  |  | Scutacaridae | 2 | 5 | 11.14 | 0.69 |
| **lower** | Amphipoda | |  |  |  |  |
| **salt marsh** |  | *Talitrus saltator* | 6 | 515 | 9.38 | 0.28 |
|  | Coleoptera | |  |  |  |  |
|  |  | *Dicheirotrichus gustavii* | 1 | 808 | 13.55 | 0.00 |
|  |  | *Dicheirotrichus* sp. | 2 | 110 | 6.42 | 0.28 |
|  | Collembola | |  |  |  |  |
|  |  | *Archisotoma besselsi* | 2 | 11 | 11.61 | 0.83 |
|  |  | *Halisotoma maritima* | 5 | 5 | 5.75 | 2.34 |
|  |  | *Thalassaphorura debilis* | 3 | 18 | 10.39 | 1.67 |
|  | Diptera | |  |  |  |  |
|  |  | Scatopsidae larvae | 3 | 20 | 10.01 | 0.30 |
|  |  | Stratiomyidae larvae | 3 | 22 | 10.33 | 1.72 |
|  | Gastropoda | |  |  |  |  |
|  |  | *Assiminea grayana* | 3 | 325 | 8.91 | 0.83 |
|  | Hemiptera | |  |  |  |  |
|  |  | *Prokelisia marginata* | 2 | 30 | 10.82 | 0.93 |
|  |  | *Orthezia urticae* | 2 | 446 | 7.41 | 0.77 |
|  | Mesostigmata | |  |  |  |  |
|  |  | *Dendrolaelaps halophilus* | 3 | 27 | 16.95 | 0.06 |
|  |  | *Pseudoparasitus germaniscus* | 3 | 22 | 15.23 | 0.71 |
|  |  | *Macrochetes* sp. juvenile | 3 | 18 | 14.93 | 0.50 |
|  |  | *Uropoda repleta* | 4 | 24 | 14.25 | 0.52 |
|  | Oribatida | |  |  |  |  |
|  |  | *Ameronothrus schneideri* | 3 | 22 | 6.06 | 0.72 |
|  |  | *Hermannia pulchella* | 9 | 73 | 7.47 | 1.03 |
| **pioneer** | Coleoptera | |  |  |  |  |
| **zone** |  | *Dicheirotrichus gustavii* | 1 | 1005 | 9.96 | 0.00 |
|  | Collembola | |  |  |  |  |
|  |  | *Archisotoma besselsi* | 4 | 20 | 8.27 | 1.58 |
|  |  | *Thalassaphorura debilis* | 2 | 20 | 12.63 | 0.27 |
|  | Diptera | |  |  |  |  |
|  |  | Dolichopodidae larvae | 3 | 21 | 11.04 | 0.68 |
|  | Hemiptera | |  |  |  |  |
|  |  | *Prokelisia marginata* | 10 | 53 | 9.54 | 1.43 |
|  | Oribatida | |  |  |  |  |
|  |  | *Ameronothrus* sp. juvenil | 3 | 15 | 6.29 | 0.81 |

**Table C**

| **dominant** | |  | **replicates** | **δ^13^C (‰)** | | **δ^15^N (‰)** | |
| --- | --- | --- | --- | --- | --- | --- | --- |
| **mesofauna taxa** | | **abbreviation** | ***n*** | **mean** | **SD** | **mean** | **SD** |
| Collembola | | | | | | | |
|  | *Archisotoma besselsi* | *ArchBess* | 6 | -24.34 | 8.77 | 9.94 | 2.36 |
|  | *Archisotoma theae* | *ArchThea* | 2 | -20.71 | 0.02 | 14.36 | 0.07 |
|  | *Entomobrya lanuginosa* | *EntmLang* | 3 | -21.97 | 0.77 | 8.55 | 0.38 |
|  | *Halisotoma maritima* | *HalsMart* | 8 | -19.11 | 1.81 | 9.36 | 5.11 |
|  | *Isotoma riparia* | *IsotRipr* | 2 | -20.54 | 0.24 | 11.66 | 0.70 |
|  | *Isotoma viridis* | *IsotVird* | 3 | -19.93 | 0.54 | 9.13 | 3.64 |
|  | *Lepidocyrtus violaceus* | *LepdViol* | 3 | -21.35 | 0.36 | 9.76 | 3.09 |
|  | *Thalassaphorura debilis* | *ThalDebl* | 7 | -20.35 | 6.07 | 12.23 | 1.67 |
| Mesostigmata | | | | | | | |
|  | *Dendrolaelaps halophilus* | *DendHalp* | 3 | -17.63 | 0.86 | 16.95 | 0.06 |
|  | *Hypoaspis aculeifer* | *HypoAcul* | 3 | -21.37 | 0.22 | 16.36 | 0.43 |
|  | *Hypoaspis praesternalis* | *HypoPrae* | 2 | -22.76 | 0.86 | 19.16 | 2.01 |
|  | *Lysigamasus* cf. *resinae* | *LysgResi* | 2 | -19.99 | 0.21 | 16.86 | 1.16 |
|  | *Lysigamasus runcatellus* | *LysgRuna* | 3 | -21.03 | 0.38 | 17.48 | 0.88 |
|  | *Lysigamasus runciger* | *LysgRuni* | 3 | -21.00 | 0.55 | 15.96 | 0.53 |
|  | *Macrochetes* sp. juvenile | *MacrSpJuv* | 3 | -16.37 | 1.50 | 14.93 | 0.50 |
|  | *Pachylaelaps longicrinitus* | *PachLong* | 2 | -22.00 | 1.14 | 17.89 | 0.63 |
|  | *Pergamasus crassipes* | *PergCras* | 3 | -20.54 | 0.75 | 15.96 | 1.76 |
|  | *Rhodocarus salarius* | *RhodSalr* | 3 | -20.66 | 0.48 | 18.22 | 0.71 |
|  | *Pseudoparasitus germaniscus* | *PseuGerm* | 3 | -17.47 | 0.22 | 15.23 | 0.71 |
|  | *Uropoda repleta* | *UropRepl* | 8 | -18.53 | 2.47 | 16.49 | 3.16 |
| Oribatida | | | | | | | |
|  | *Ameronothrus schneideri* | *AmerSchn* | 3 | -15.05 | 0.64 | 6.06 | 0.72 |
|  | *Banksinoma lanceolata* | *BankLanc* | 4 | -21.59 | 0.78 | 15.00 | 0.46 |
|  | *Dissorhina ornata* | *DissOrnt* | 3 | -22.88 | 1.01 | 13.57 | 0.62 |
|  | *Hermannia pulchella* | *HermPulc* | 9 | -17.30 | 0.52 | 7.47 | 1.03 |
|  | *Liebstadia similis* | *LiebSiml* | 4 | -21.50 | 0.33 | 10.26 | 1.78 |
|  | *Microppia minus* | *MicrMins* | 3 | -20.58 | 0.15 | 14.39 | 0.96 |
|  | *Ramusella clavipectinata* | *RamsClav* | 2 | -22.27 | 1.12 | 14.59 | 0.34 |
|  | *Scheloribates laevigatus* | *SchlLaev* | 3 | -21.87 | 0.15 | 12.82 | 1.21 |

**Table D**

| **salt marsh** | **basal food** | | **replicates** | **type of** | **δ^13^C (‰)** | |
| --- | --- | --- | --- | --- | --- | --- |
| **zone** | **resources** | | ***n*** | **CO_2_-fixation** | **mean** | **SD** |
| **upper** | vascular plants | |  |  |  |  |
| **salt marsh** |  | *Atriplex littoralis* | 3 | C_3_ plant | -27.34 | 0.04 |
|  |  | *Atriplex prostrata* | 3 | C_3_ plant | -29.31 | 0.02 |
|  |  | *Elytrigia atherica* | 3 | C_3_ plant | -25.44 | 0.09 |
|  | OM - organic matter | | 3 |  | -25.90 | 0.76 |
| **lower** | vascular plants | |  |  |  |  |
| **salt marsh** |  | *Atriplex portulacoides* | 3 | C_3_ plant | -27.13 | 0.05 |
|  |  | *Puccinellia maritima* | 3 | C_3_ plant | -24.84 | 0.06 |
|  |  | *Salicornia europaea* | 3 | C_3_ plant | -28.08 | 0.05 |
|  |  | *Spergularia salina* | 3 | C_3_ plant | -26.38 | 0.06 |
|  |  | *Triglochin maritima* | 3 | C_3_ plant | -25.12 | 0.00 |
|  | OM - organic matter | | 3 |  | -29.90 | 0.26 |
| **pioneer** | vascular plants | |  |  |  |  |
| **zone** |  | *Limonium vulgare* | 3 | C_3_ plant | -25.05 | 0.14 |
|  |  | *Salicornia stricta* | 3 | C_3_ plant | -28.02 | 0.04 |
|  |  | *Spartina anglica* | 3 | C_4_ plant | -13.17 | 0.05 |
|  |  | *Suaeda maritima* | 6 | C_3_ plant | -28.35 | 0.05 |
|  | macroalgae | |  |  |  |  |
|  |  | *Ceramium* sp. | 2 | C_4_ algae | -17.77 | 0.09 |
|  |  | *Chaetomorpha linum* | 3 | C_4_ algae | -18.17 | 2.88 |
|  |  | *Enteromorpha* sp. | 3 | C_4_ algae | -18.21 | 0.72 |
|  |  | *Fucus vesiculosus* | 3 | C_3_ algae | -23.34 | 0.18 |
|  |  | *Rhizoclonium riparium* | 3 | C_4_ algae | -16.90 | 2.57 |
|  |  | *Ulva lactuca* | 2 | C_4_ algae | -13.89 | 0.00 |
|  | OM - organic matter | | 2 |  | -13.45 | 0.06 |

**Table E**

| **salt marsh** | **mesofauna consumer** | | r**eplicates** | **δ^15^N (‰)** | | **δ^13^C (‰)** | | **food sources (%)** | | |
| --- | --- | --- | --- | --- | --- | --- | --- | --- | --- | --- |
| **zone** | **taxa** | | ***n*** | **mean** | **SD** | **mean** | **SD** | **C3** | **C4** | **OM** |
| **upper** | Collembola | |  |  |  |  |  |  |  |  |
| **salt marsh** |  | *Entomobrya lanuginosa* | 3 | 4.22 | 0.38 | -24.79 | 0.77 | 60 | 0 | 40 |
|  |  | *Halisotoma maritima* | 3 | 8.65 | 1.47 | -23.21 | 0.10 | 55 | 0 | 45 |
|  |  | *Isotoma riparia* | 2 | 7.33 | 0.70 | -23.36 | 0.24 | 31 | 0 | 69 |
|  |  | *Isotoma viridis* | 3 | 4.80 | 3.64 | -22.75 | 0.54 | 55 | 0 | 45 |
|  |  | *Lepidocyrtus violaceus* | 3 | 5.43 | 3.09 | -24.17 | 0.36 | 36 | 0 | 64 |
|  |  | *Thalassaphorura debilis* | 2 | 9.33 | 1.87 | -22.20 | 0.40 | 74 | 0 | 26 |
|  |  | **mean** |  |  |  |  |  | **52** | **0** | **48** |
|  | Oribatida | |  |  |  |  |  |  |  |  |
|  |  | *Liebstadia similis* | 4 | 5.93 | 1.78 | -24.32 | 0.33 | 30 | 0 | 70 |
|  |  | *Scheloribates laevigatus* | 3 | 8.49 | 1.21 | -24.69 | 0.15 | 35 | 0 | 65 |
|  |  | **mean** |  |  |  |  |  | **33** | **0** | **68** |
| **lower** | Collembola | |  |  |  |  |  |  |  |  |
| **salt marsh** |  | *Archisotoma besselsi* | 2 | 12.92 | 0.83 | -24.96 | 1.02 | 77 | 0 | 23 |
|  |  | *Halisotoma maritima* | 5 | 7.06 | 2.34 | -24.66 | 1.22 | 78 | 0 | 22 |
|  |  | *Thalassaphorura debilis* | 3 | 11.70 | 1.67 | -21.65 | 1.11 | 90 | 0 | 10 |
|  |  | **mean** |  |  |  |  |  | **82** | **0** | **18** |
|  | Oribatida | |  |  |  |  |  |  |  |  |
|  |  | *Ameronothrus schneideri* | 3 | 7.37 | 0.72 | -21.87 | 0.64 | 96 | 0 | 4 |
|  |  | *Hermannia pulchella* | 9 | 8.78 | 1.03 | -24.12 | 0.52 | 90 | 0 | 10 |
|  |  | **mean** |  |  |  |  |  | **93** | **0** | **7** |
| **pioneer** | Collembola | |  |  |  |  |  |  |  |  |
| **zone** |  | *Archisotoma besselsi* | 4 | 11.29 | 1.58 | -20.91 | 1.66 | 56 | 23 | 21 |
|  |  | *Thalassaphorura debilis* | 2 | 15.65 | 0.27 | -17.20 | 0.68 | 25 | 37 | 38 |
|  |  | **mean** |  |  |  |  |  | **41** | **30** | **30** |
|  | Oribatida | |  |  |  |  |  |  |  |  |
|  |  | *Ameronothrus* sp. juvenil | 3 | 9.31 | 0.81 | -19.14 | 1.25 | 72 | 13 | 15 |
|  |  | **mean** |  |  |  |  |  | **72** | **13** | **15** |
